# Joint Optimization of Deep Neural Network-Based Dereverberation and Beamforming for Sound Event Detection in Multi-Channel Environments

**DOI:** 10.3390/s20071883

**Published:** 2020-03-28

**Authors:** Kyoungjin Noh, Joon-Hyuk Chang

**Affiliations:** Department of Electronics and Computer Engineering, Hanyang University, Seoul 04763, Korea; nkj0318@hanyang.ac.kr

**Keywords:** sound event detection, dereverberation, acoustic beamforming, convolutional recurrent neural network, joint optimization

## Abstract

In this paper, we propose joint optimization of deep neural network (DNN)-supported dereverberation and beamforming for the convolutional recurrent neural network (CRNN)-based sound event detection (SED) in multi-channel environments. First, the short-time Fourier transform (STFT) coefficients are calculated from multi-channel audio signals under the noisy and reverberant environments, which are then enhanced by the DNN-supported weighted prediction error (WPE) dereverberation with the estimated masks. Next, the STFT coefficients of the dereverberated multi-channel audio signals are conveyed to the DNN-supported minimum variance distortionless response (MVDR) beamformer in which DNN-supported MVDR beamforming is carried out with the source and noise masks estimated by the DNN. As a result, the single-channel enhanced STFT coefficients are shown at the output and tossed to the CRNN-based SED system, and then, the three modules are jointly trained by the single loss function designed for SED. Furthermore, to ease the difficulty of training a deep learning model for SED caused by the imbalance in the amount of data for each class, the focal loss is used as a loss function. Experimental results show that joint training of DNN-supported dereverberation and beamforming with the SED model under the supervision of focal loss significantly improves the performance under the noisy and reverberant environments.

## 1. Introduction

Sound event detection (SED) is desired as a task that detects the onset and offset times for each sound event in an audio segment. Various sounds always occur around us, and SED enables many services, including social care [1], audio surveillance [2,3], drone detection [4], and bird detection [5], by allowing machines to recognize sound events like the human auditory system. In recent years, the Detection and Classification of Acoustic Scenes and Events (DCASE) challenge and deep learning have significantly accelerated the research of SED. In the first DCASE challenge held in 2013, all proposed algorithms were based on shallow learning such as the hidden Markov model (HMM), support vector machine (SVM), and the Gaussian mixture model (GMM). In DCASE 2013, only a small number of teams participated, and the performances of the systems turned out not to be desirable [6]. Since the deep neural network (DNN)-based polyphonic SED algorithm was proposed in 2015 [7], deep learning-based SED studies have begun to pour out with the DCASE challenge (2016, 2017, 2018, 2019). In particular, deep learning structures based on the convolutional neural network (CNN) [8,9], recurrent neural network (RNN) [10,11,12], and convolutional recurrent neural network (CRNN) [13] showed the state-of-the-art performance, and data augmentation methods were proposed to maximize the learning ability of deep learning models [14]. Since the CNN can extract optimized features with trainable convolutional filters, it achieves a better performance than image-like inputs such as the log-scale Mel filter bank (LMFB), which is most used in the SED domain. Furthermore, the RNN, which can remember previous inputs through time, also performs well due to the time-series characteristics of the audio signal. Recently, the CNN and CRNN models with additional techniques, including the modified CNN [15] and pooling methods [16], were proposed. Furthermore, further studies combined with other tasks such as sound event detection and segmentation using the weakly labeled data [17] and joint sound event detection and localization [18] were proposed.

In contrast, in the speech recognition domain, the use of the LMFB as a feature vector and the CNN, RNN, and CRNN as the classifiers for the acoustic model are similar to the SED, but studies of integrating a preprocessor, such as acoustic beamforming or dereverberation with the acoustic model using multi-channel audio signals, have been actively conducted to improve recognition accuracy [19,20,21]. Furthermore, the joint optimization method on DNN-supported dereverberation and beamforming with an end-to-end speech recognition model was recently proposed [22]. Similarly, the joint optimization onto DNN-supported dereverberation and beamforming with the x-vector net was introduced in the speaker verification domain [23]. However, unlike speech recognition and speaker verification, there have not been many studies to enhance the audio signals for the SED because it is challenging to distinguish the evident audio from ambient noise due to the wide variety of target sounds. Sometimes, audio enhancement even degrades the SED performance, so some studies have been conducted on a limited basis with a weak level of noise reduction [24] or adaptive noise reduction [25] for the SED. Additionally, when using multi-channel audio signals, only studies using binaural features [26] or spatial features [27] and classification with the 3D CNN [28] have been reported, rather than combining them with preprocessing algorithms. Nevertheless, research on the combination of the preprocessor and the SED to take advantage of the multi-microphone has to be carried out for further performance enhancement.

In this paper, we propose a joint optimization method on DNN-supported dereverberation and beamforming for the SED under noisy and reverberant conditions. Because deep learning seamlessly optimizes beamforming and dereverberation through training, it is possible to combine beamforming and dereverberation with the SED to obtain optimized overall performance. One significant contribution compared to previous studies is the effectiveness of the cascade of DNN-supported weighted prediction error (WPE) dereverberation, the DNN-supported minimum variance distortionless response (MVDR) beamformer, and the SED network. Further, we present jointly training the final objective, the cost function, of the SED task. Furthermore, we employ the focal loss [29] within this task, since it is challenging to equalize the data amount of each sound class because the audio lengths of each class are all different in reality. Specifically, a mini-batch balancing method [30] in the training process is proposed to overcome this problem, but focal loss further helps to compensate this problem naturally in the training process. The evaluation was conducted based on the Tampere University (TAU) Spatial Sound Events 2019 dataset (http://dcase.community/challenge2019/task-sound-event-localization-and-detection), which showed significant improvement compared to conventional methods in the F-score and error rate.

Section 2 describes the proposed system, which is composed of the DNN-supported WPE dereverberation, DNN-supported MVDR beamformer, and SED. The dataset, evaluation metrics, experimental setup, and results are described in Section 3. Finally, conclusions are provided in Section 4.

## 2. Proposed System Overview

**Figure 1:**
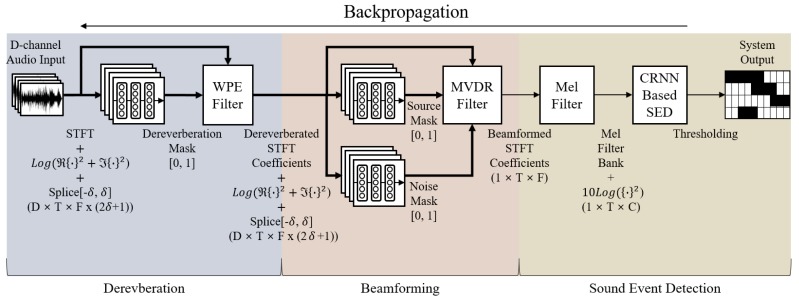
Block diagram of the proposed system. WPE, weighted prediction error; MVDR, minimum variance distortionless response; SED, sound event detection.

In this section, we fully describe our proposed system, which consists of three parts, as depicted in Figure 1. The first part of the system is designed for dereverberation by the DNN-supported WPE using multi-channel signals (in Section 2.1). In the second part, the DNN-supported MVDR beamforming is performed using the multi-channel output of the first part, and the single-channel beamformed signal is estimated as a result. Finally, the CRNN based SED assesses the presence or absence of sound events, including the onset and offset detection. Then, all the parts of the system are jointly optimized with the focal loss as a loss function. The details of each part of the proposed system are described in the following subsections.

### 2.1. DNN-Supported WPE Dereverberation

This subsection explains in detail the DNN-supported WPE dereverberation part including classical WPE dereverberation and DNN-supported WPE dereverberation reported in [20,21,22,23]. When we observe a signal using *D* microphones in a noisy and reverberant environment, the observed signal $y_{t,f,d}$ can be represented in the short-time Fourier transform (STFT) domain as follows:

(1) $$y_{t,f,d}=x_{t,f,d}^{\left(\mathrm{early}\right)}+x_{t,f,d}^{\left(\mathrm{late}\right)}+n_{t,f,d}$$

where $x_{t,f,d}^{\left(\mathrm{early}\right)}$, $x_{t,f,d}^{\left(\mathrm{late}\right)}$, and $n_{t,f,d}$ denote the source signal convolved with the early part of the room impulse response (RIR) and with the late reflection and noise signal, respectively. Furthermore, *t* is the time frame index; *f* is the frequency bin index; and *d* is the microphone channel index, respectively. We assume that the first 50 ms after the main peak of the RIR contributes to the early reflection, and the remaining part becomes the late reflection. The purpose of dereverberation is to subtract late reflection components from the observed signal as follows:

(2) $${\hat{x}}_{t,f,d}^{\left(\mathrm{early}\right)}=y_{t,f,d}-G_{f,d}^{H}{\tilde{y}}_{t-\Delta ,f}$$

where $G_{f,d}^{H}$, ${\tilde{y}}_{t-\Delta ,f}$, and Δ are the stacked representations of the linear prediction (LP) filter (WPE filter in Figure 1), the observation, and a delay for LP, respectively. To estimate the early reflection component, the classical WPE algorithm finds the LP filter based on the maximum likelihood (ML) for which the WPE assumes that the desired signal follows a zero-mean complex Gaussian distribution with a time-varying variance $\lambda_{t,f}$. There is no closed-form solution of the ML optimization problem, but an iterative procedure alternates between estimating the filter coefficients $G_{f,d}^{H}$ and the time-varying variance $\lambda_{t,f}$ to find $G_{f,d}^{H}$ as follows:

(3) $$\lambda_{t,f}=\frac{1}{(\delta +1+\delta )D}\sum_{i=t-\delta }^{t+\delta }\sum_{d}{|{\hat{x}}_{t,f,d}^{\left(\mathrm{early}\right)}|}^{2}$$

(4) $$R_{f}=\sum_{t}\frac{{\tilde{y}}_{t-\Delta ,f}{\tilde{y}}_{t-\Delta ,f}^{H}}{\lambda_{t,f}}\;\;\;\in C^{DK\times DK}$$

(5) $$P_{f}=\sum_{t}\frac{{\tilde{y}}_{t,f}y_{t-\Delta ,f}^{H}}{\lambda_{t,f}}\;\;\;\;\in C^{DK\times D}$$

(6) $$G_{f}=R_{f}^{-1}P_{f}\;\;\;\;\;\;\;\in C^{DK\times D}$$

where (δ+1+δ) means the number of context frames to improve the variance estimate, $R_{f}$ is the correlation matrix, $P_{f}$ is the correlation vector, and *K* is the order of the LP filter. DNN-supported WPE dereverberation replaces the iterative procedure to estimate power spectrum ${|{\hat{x}}_{t,f,d}^{\left(\mathrm{early}\right)}|}^{2}$ in Equation (3). For this, we estimate the masks for calculating the desired power spectrum ${|{\hat{x}}_{t,f,d}^{\left(\mathrm{early}\right)}|}^{2}$ from the given input power spectrum ${|y_{t,f,d}|}^{2}$. Specifically, the log-scale power spectra (LPS) $y_{t,f,d}$ are used as the input of the DNNs, which use ReLU with max clamp of one for the activation function of the output layer so as to limit the estimated masks within [0,1]. We can calculate ${|{\hat{x}}_{t,f,d}^{\left(\mathrm{early}\right)}|}^{2}$ with ${|y_{t,f,d}|}^{2}$ and estimated masks. Finally, ${\hat{x}}_{t,f,d}^{\left(\mathrm{early}\right)}$ is estimated by following sequence (3)→(4)→(5)→(6)→(2), and it is tossed as the input of the DNN-supported MVDR beamformer part. Since the range of the masks is bounded within [0,1], the DNN is easier to optimize than the direct prediction method of the desired power spectrum when jointly training the full networks [22].

### 2.2. DNN-Supported MVDR Beamformer

Originally, the MVDR beamformer used the steering vector, which depends on the angle of the desired signal from the source to minimize the residual noise while constraining the distortion of the signal. The steering vector can be obtained from an estimate of the direction of arrival (DoA) and the optimal signal is calculated by inducing the maximum beam gain in the steering vector direction and the minimum beam gain in the remaining direction. However, the MVDR beamformer also can be derived by speech and noise power spectral density (PSD) matrices without the steering vector. According to [31], the enhanced single-channel output ${\hat{x}}_{t,f}$ can be found by multiplying the gain $H_{MVDR}^{H}$ (MVDR filter in Figure 1) by the observed multi-channel input signal $y_{t,f}$ as follows:

(7) $$H_{MVDR}^{H}=\frac{\Phi_{nn}^{-1}\Phi_{xx}}{tr(\Phi_{nn}^{-1}\Phi_{xx})}u\;\;\;\in C^{D}$$

(8) $${\hat{x}}_{t,f}=H_{MVDR}^{H}y_{t,f}$$

where $\Phi_{xx}$ and $\Phi_{nn}$ respectively denote the PSD matrices of the source and noise components and **u** is a one-hot vector for the reference microphone. In addition, tr means the trace of the matrix. In the DNN-supported MVDR beamformer, similar to the DNN-supported WPE dereverberation, two networks are separately trained for estimating masks in calculating the source and noise PSD matrices, where *v* denotes the signal attribute and $\theta_{f}$ is a predefined decision threshold, respectively [19,20,22,23]. These masks are averaged over the microphone channel *d*. As a result, the PSD matrices of the source and noise are found as follows:

(9) $$\Phi_{vv}=\left.\sum_{t}{\hat{M}}_{t,f}^{\left(v\right)}y_{t,f}y_{t,f}^{H}/\sum_{t}{\hat{M}}_{t,f}^{\left(v\right)}\;\;v\in \left\{x,n\right\}\right.$$

where ${\hat{M}}_{t,f}^{\left(v\right)}\in \left[0,1\right]$ denotes the estimated time-frequency mask calculated by the DNN, which uses the sigmoid as the activation function of the output layer. Finally, single-channel beamformed STFT coefficients ${\hat{x}}_{t,f}$ are estimated by following order (9)→(7)→(8). Then, ${\hat{x}}_{t,f}$ is conveyed to the SED model for predicting sound events.

### 2.3. Sound Event Detection

**Figure 2:**
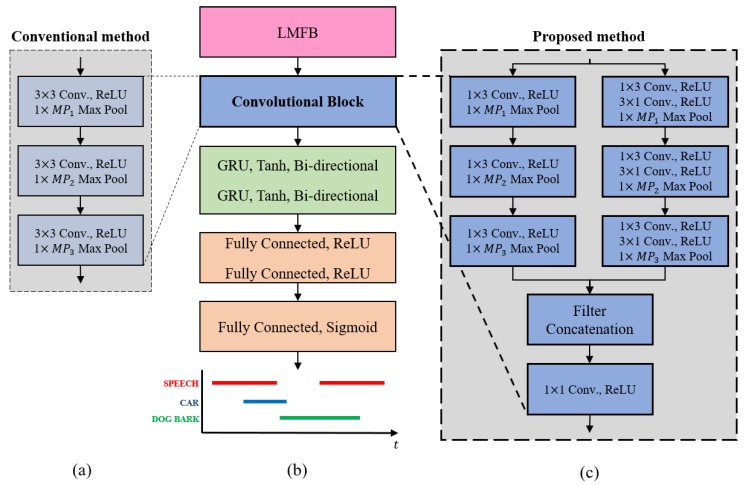
Overview of the SED model: (**a**) convolutional block of the Detection and Classification of Acoustic Scenes and Events (DCASE) 2019 Task 3 baseline [18]; (**b**) CRNN-based SED model; (**c**) proposed convolutional block. LMFB, log-scale Mel filter bank.

For the SED, the LMFB is used as an input feature, which can be calculated by multiplying the magnitude spectrum with the Mel filter and then taking the logarithm. The input features are normalized using the global mean and variance statistics before being fed to the CRNN-based SED model, which is illustrated in Figure 2. Figure 2a–c show the CRNN-based SED model, the conventional convolutional block of the DCASE 2019 Task 3 baseline [18], and the proposed convolutional block, respectively. Unlike the conventional method using the three layers of the 3 × 3 convolution filter, the proposed convolutional block consists of two parallel parts inspired by VGGNet [32] and Inception V2 [33]. The first part conducts the convolution in the direction of the frequency axis only, and the second part performs the convolution in the direction of the time-frequency axis, then the two parts are concatenated. For the second part, the 3 × 3 convolution is divided into 1 × 3 convolution and 3 × 1 convolution. Finally, 1 × 1 convolution is used to reduce the computational cost. The output of the convolutional block is fed to the two layers of the bi-directional gated recurrent unit (GRU) RNN. Next, the output of the bi-directional GRU is connected to the fully connected layers and the output layer with the sigmoid function as an activation function, so that the value of the outputs is selected between zero and one for each class.

### 2.4. Joint Optimization

This section summarizes and explains how the DNN-supported dereverberation, beamforming, and the CRNN-based SED models are organized into a cascaded network. First, when the D-channel audio signal is input, the magnitudes of the STFT coefficients are calculated and then fed to the DNN. The DNN estimates the dereverberation mask, and then the magnitudes of the STFT coefficients of the dereverberated signal can be calculated using Equations (2), (3) and (6). Next, this output is fed into another DNN to estimate the source and noise masks for the neural MVDR beamformer. Using Equations (7)–(9), the magnitudes of the STFT coefficient of the single-channel enhanced signal are obtained. Then, multiplying these values with the Mel filters, the LMFB is calculated, which serves as an input for the CRNN-based SED model. The whole network is trained by the loss, which is calculated with the label and the SED output. At this time, the focal loss is considered as a loss function for further improving the performance. As for the SED, equalizing the data amount of each class is challenging because the audio lengths of each class are all different. The focal loss is useful for compensating for this problem naturally when training the deep learning model by giving a stronger loss to those that fail to estimate [29]. The focal loss is defined as follows:

(10) $$\mathrm{FL}\left(p_{t}\right)=-{\left(1-p_{t}\right)}^{\gamma }\log\left(p_{t}\right)$$

(11) $$p_{t}=\left\{\begin{array}{cc} p, & \mathrm{if}\;gt=1\\ 1-p, & \mathrm{otherwise} \end{array}\right.$$

where gt represents the ground truth, p∈[0,1] is the model’s estimated probability, and γ denotes the tunable focusing parameter.

All of the processes described above are differentiable, so the backpropagation with the chain rule is possible. Motivated by this, in the end, we perform joint training for the cascaded architecture of DNN-supported WPE dereverberation, the DNN-supported MVDR beamformer, and the SED network according to the focal loss, as depicted in Figure 2.

As for the joint optimization, we demand complex-valued operations including the complex-valued inverse in Equations (6) and (7). As in [19,23], the complex-valued operations using real-valued operations are implemented by separately computing real and imaginary parts. When C is a complex-valued matrix and **A** and **B** are real-valued matrices corresponding to real and imaginary parts, C can be expressed as C=A+iB. At this time, the complex-valued matrix inverse operations can be calculated as follows [34]:

(12) $$ℜ\left(C^{-1}\right)={\left(A+{\mathrm{BA}}^{-1}B\right)}^{-1}$$

(13) $$ℑ\left(C^{-1}\right)=-{\left(A+{\mathrm{BA}}^{-1}B\right)}^{-1}{\mathrm{BA}}^{-1}$$

## 3. Experiments and Results

### 3.1. Dataset

The proposed algorithm was evaluated with the TAU Spatial Sound Events 2019 dataset. The dataset consists of the two datasets, Ambisonic and Microphone Array [35]. The TAU Spatial Sound Events 2019-Ambisonic dataset provides four-channel first-order ambisonic (FOA) recordings, while the TAU Spatial Sound Events 2019-Microphone Array dataset provides four-channel directional microphone recordings from a tetrahedral array configuration. Each dataset consists of 500 audio files, 400 for development and 100 for evaluation. The records are one minute long, the sampling frequency 48,000 Hz, and the signal-to-noise ratio (SNR) for sound events and ambient noise 30 dB. These recordings were synthesized using the spatial room impulse response (IRs) collected from five indoor locations at 504 unique combinations of azimuth-elevation-distance. The collected IRs were convolved with the DCASE 2016 Task 2 dataset. In the DCASE 2016 Task 2 dataset, there are 11 classes of sound events such as clearing throat, coughing, door knock, door slam, drawer, laughter, keyboard, keys (putting on table), page-turning, phone ringing, and speech, and each class consists of 20 audio files. Finally, each development dataset was divided into four cross-validation [36] splits of 100 recordings each. Additionally, to consider the noisy environment, we mixed the datasets with the ambient noise recorded at an indoor location inside the Hanyang University campus in Seoul, Korea, under 10 dB SNR. Two simple data augmentation methods (pitch shifting [14] and block mixing [10] using monophonic audio clips) were applied in the training process for the model generalization to reduce overfitting.

### 3.2. Evaluation Metrics

To evaluate the performance of the SED model, we measured the segment-based F-score and error rate (ER) in the same way as the DCASE 2019 Task 3. The F-score and ER were calculated in segments of one second with no overlap [37,38]. Therefore, the labels and the SED outputs were generated on average for segments of one second to calculate metrics. First, the F-score, which measures the effectiveness of retrieval, is calculated as follows:

(14) $$F=\frac{2\cdot \sum_{k=1}^{K}TP}{2\cdot \sum_{k=1}^{K}TP+2\cdot \sum_{k=1}^{K}FP+2\cdot \sum_{k=1}^{K}FN}$$

where *K* is the number of segments and TP(k) denotes the number of true positives, which is the total number of sound event classes that were active in both the reference and predictions for the segment. In addition, FP(k) denotes the number of false positives, which is the number of sound event classes that were active in the prediction, but were inactive in the reference. Similarly, FN(k) is the number of false negatives, which is the number of sound event classes inactive in the predictions, but active in the reference. Additionally, the ER, which measures the amount of errors, is given as follows:

(15) $$ER=\frac{\sum_{k=1}^{K}S\left(k\right)+\sum_{k=1}^{K}D\left(k\right)+\sum_{k=1}^{K}I\left(k\right)}{\sum_{k=1}^{K}N\left(k\right)}$$

where N(k) is the total number of active sound event classes in the reference. In addition, S(k), D(k), and I(k) are called the substitution, deletion, and insertion, respectively, which are mathematically defined as:

(16) S(k)=min(FN(k),FP(k)),

(17) D(k)=max(0,FN(k)−FP(k)),

(18) I(k)=max(0,FP(k)−FN(k)).

As for the ideal case, it is noted that the F-score and ER become one and zero, respectively.

### 3.3. Experimental Setup

The evaluation was performed with a window length of 40 ms, a hop length of 20 ms, and a fast Fourier transform (FFT) size of 2048 points. Therefore, we obtained 3000 frames in one file since the file was 60 seconds long, and the input sequence length *T* for training was 128. For dereverberation and beamforming, the multi-layer perceptron, which consisted of three hidden layers with 1024 nodes, was used. ReLU was chosen for the activation function at the hidden layers. For the DNN input, we used the log-scale power spectra (folded frequency bins were discarded) as features that were spliced with three left and three right context frames. Note that the parameters of the LP filter for the WPE were fixed to (Δ,K)=(3,10). For sound event detection, first, the number of Mel filters for LMFB *C* was 240. Next, the number of CNN filters for each layer was [64, 64, 64], and the max pooling sizes along the frequency axis ($MP_{1}$, $MP_{2}$, and $MP_{3}$) were 6, 5, and 4, respectively. Additionally, the size of two GRU layers and two fully connected (FC) layers was [128, 128] and [256, 256], and the drop-out rate for the FC layers was 0.5. We summarize the configurations of the neural networks in Table 1 and Table 2. The batch size was 16, and an early stopping method was applied. Batch normalization [39] was applied to all networks, and the networks were optimized by Adam [40]. The focus parameter γ of the focal loss was set to two.

### 3.4. Results

Table 3 and Table 4 show the results with the TAU Spatial Sound Events 2019-Ambisonic development dataset and TAU Spatial Sound Events 2019-Microphone Array development dataset, respectively. First, by replacing the convolutional block, the F-score increased by approximately 1.6% on average compared to the conventional method in both datasets, and the ER also improved to 0.05. This result exhibited that using the different types of blocks in the convolutional block to extract features and concatenate them also worked well for the SED. Next, the performance was improved in all cases where the WPE was combined with the SED, the MVDR was combined with the SED, and the WPE and MVDR were connected with the SED and then jointly trained, respectively. The one point of these results was that MVDR was much more useful than WPE. However, this may be because the reverberation of the dataset was not active. Finally, the focal loss also turned out to be helpful in gaining the performances for the unbalanced dataset. The performance of Split 2, which had a slightly lower performance than the other splits, was relatively increased. Subsequently, the average F-score increased by 13.1%, and the ER improved 0.23 compared to the conventional method. For the DCASE 2019 Task 3 challenge results, two systems showed better performance than our proposed system with this dataset, and they achieved the F-score of 98.2%, while Xue_JDAI_task3_1 [41] achieved the F-score of 93.4%. However, MazzonYasuda_NTT_task3_3 [42] used 134M parameters for a vast ensemble model because the DCASE 2019 challenge did not require limited complexity. In contrast, the number of parameters in our system was 21M only. Table 5 and Table 6 show the results at 10 dB SNR for the Ambisonic and Microphone Array development datasets, respectively. Similar to the original 30 dB datasets, the performance in the noisy environment was also improved in all cases where the WPE was combined with the SED, the MVDR was combined with the SED, and the WPE and MVDR were attached to the SED and then jointly trained, respectively. Table 7 shows the F-score and ER results of the evaluation dataset. Compared to the DCASE 2019 Task 3 algorithms, the proposed algorithm showed 4% better performance under the 10 dB SNR environment.

## 4. Conclusions

The CRNN-based SED model, which combines the DNN-supported WPE dereverberation and the DNN-supported MVDR beamformer, was jointly trained using a single loss function. Since the DNN-supported WPE dereverberation and MVDR beamformer were all differentiable, the gradients derived from the SED part could be backpropagated to update all the parameters of the DNN-supported dereverberation and beamforming. As for the loss function, we used the focal loss to compensate for the imbalance in the amount of data between classes. Experimental results showed that the joint training and focal loss improved the F-score and error rate of the SED, especially noisy environments.

## Figures and Tables

**Table 1:** Configuration of DNNs for dereverberation and the beamformer.

Layers	Output size (Channels × Frames × Frequency Bins)
Input (Log power spectra)	4 × 128 × (1025 × 7) (Including left and right context frames)
Hidden Layer 1	4 × 128 × 1024
Hidden Layer 2	4 × 128 × 1024
Hidden Layer 3	4 × 128 × 1024
Output (Mask)	4 × 128 × 1025

**Table 2:** Configuration of CRNN.

Layers	Output Size
Input (Log Mel filter bank)	1 × 128 × 240 (Feature maps × Frames × Mel bins)
Convolutional Layer 1	64 × 128 × 40/64 × 128 × 40
Convolutional Layer 2	64 × 128 × 8/64 × 128 × 8
Convolutional Layer 3	64 × 128 × 2/64 × 128 × 2
Concatenate	128 × 128 × 2
1 × 1 Convolution	64 × 128 × 2
GRU Layer 1	128 × 128
GRU Layer 2	128 × 128
Fully Connected Layer 1	128 × 256
Fully Connected Layer 2	128 × 256
Output	128 × 11 (frames × classes)

**Table 3:** F-score and error rate (ER) results on the TAU Spatial Sound Events 2019-Ambisonic development dataset.

		DCASE 2019 Task 3 Baseline [18]	Proposed SED	Proposed WPE +SED	Proposed MVDR +SED	Proposed WPE+MVDR +SED	Proposed WPE+MVDR +SED +FL
**F-score** **(%)**	Split 1	81.2	82.8	83.0	89.6	91.7	92.5
	Split 2	78.0	80.1	81.2	88.1	90.8	92.0
	Split 3	80.5	81.2	82.5	89.5	91.5	93.0
	Split 4	79.8	80.8	82.0	89.4	91.2	93.5
	Overall	79.9	81.2	82.2	89.2	91.3	92.8
**Error** **rate**	Split 1	0.31	0.28	0.27	0.15	0.14	0.12
	Split 2	0.37	0.32	0.31	0.17	0.16	0.14
	Split 3	0.33	0.30	0.28	0.15	0.15	0.13
	Split 4	0.34	0.31	0.28	0.16	0.15	0.12
	Overall	0.34	0.30	0.29	0.16	0.15	0.13

**Table 4:** F-score and ER results on the TAU Spatial Sound Events 2019-Microphone Array development dataset.

		DCASE 2019 Task 3 Baseline [18]	Proposed SED	Proposed WPE +SED	Proposed MVDR +SED	Proposed WPE+MVDR +SED	Proposed WPE+MVDR +SED +FL
**F-score** **(%)**	Split 1	81.5	82.9	83.8	90.5	92.3	93.6
	Split 2	79.1	81.5	82.5	89.9	91.5	93.5
	Split 3	80.5	82.1	83.0	90.3	92.1	93.7
	Split 4	79.8	81.5	83.3	90.1	91.9	93.1
	Overall	80.2	82.0	83.2	90.2	92.0	93.5
**Error** **rate**	Split 1	0.31	0.28	0.25	0.15	0.14	0.08
	Split 2	0.37	0.30	0.27	0.17	0.15	0.10
	Split 3	0.35	0.29	0.27	0.16	0.14	0.09
	Split 4	0.33	0.30	0.28	0.18	0.13	0.09
	Overall	0.34	0.29	0.27	0.17	0.14	0.09

**Table 5:** F-score and ER results at 10 dB SNR: Ambisonic development dataset.

		DCASE 2019 Task 3 Baseline [18]	Proposed SED	Proposed WPE +SED	Proposed MVDR +SED	Proposed WPE+MVDR +SED	Proposed WPE+MVDR +SED +FL
**F-score** **(%)**	Split 1	69.4	70.3	72.0	79.4	82.0	82.4
	Split 2	68.2	71.5	72.4	79.0	81.2	81.1
	Split 3	70.2	72.6	74.4	80.4	83.3	83.2
	Split 4	69.6	73.5	73.1	80.2	82.4	83.0
	Overall	69.4	72.0	73.0	79.8	82.2	82.4
**Error** **rate**	Split 1	0.51	0.48	0.46	0.35	0.28	0.27
	Split 2	0.53	0.48	0.45	0.33	0.29	0.29
	Split 3	0.50	0.47	0.46	0.34	0.28	0.26
	Split 4	0.50	0.46	0.46	0.35	0.27	0.25
	Overall	0.51	0.47	0.46	0.34	0.28	0.27

**Table 6:** F-score and ER results at 10 dB SNR: Microphone Array development dataset.

		DCASE 2019 Task 3 Baseline [18]	Proposed SED	Proposed WPE +SED	Proposed MVDR +SED	Proposed WPE+MVDR +SED	Proposed WPE+MVDR +SED +FL
**F-score** **(%)**	Split 1	70.1	71.0	73.1	78.5	81.5	82.9
	Split 2	70.6	71.1	73.4	79.0	80.2	80.8
	Split 3	70.4	71.4	74.2	80.6	82.4	82.6
	Split 4	70.5	72.4	74.8	79.6	83.4	83.2
	Overall	70.4	71.5	73.9	79.4	81.9	82.4
**Error** **rate**	Split 1	0.48	0.48	0.45	0.34	0.29	0.26
	Split 2	0.51	0.49	0.47	0.37	0.33	0.30
	Split 3	0.47	0.45	0.46	0.33	0.29	0.27
	Split 4	0.46	0.45	0.43	0.32	0.26	0.24
	Overall	0.48	0.47	0.45	0.34	0.29	0.26

**Table 7:** F-score and ER results: evaluation dataset.

		30 dB SNR		10 dB SNR	
**Algorithms**		**F-Score (%)**	**ER**	**F-Score (%)**	**ER**
Kapka_SRPOL_task3_2 [43]		94.7	**0.08**	81.0	0.31
Cao_Surrey_task3_4 [44]		**95.5**	**0.08**	79.4	0.32
DCASE 2019 Task 3 baseline [18]		85.4	0.28	73.2	0.47
Proposed WPE+MVDR+SED+FL		93.3	0.11	**85.3**	**0.25**
